# A Robust Process to Produce Lignocellulosic Nanofibers from Corn Stover, Reed Canary Grass, and Industrial Hemp

**DOI:** 10.3390/polym15040937

**Published:** 2023-02-14

**Authors:** Danielle Uchimura Pascoli, Anthony Dichiara, Rick Gustafson, Renata Bura

**Affiliations:** 1 School of Environmental and Forest Sciences, University of Washington, Seattle, WA 98195, USA; 2 VERDE Nanomaterials Inc., Davis, CA 95618, USA

**Keywords:** lignocellulosic nanofibers, nanocellulose, biomass waste feedstock, corn stover, reed canary grass, industrial hemp, material properties, characterization

## Abstract

The use of agricultural waste biomass for nanocellulose production has gained interest due to its environmental and economic benefits compared to conventional bleached pulp feedstock. However, there is still a need to establish robust process technologies that can accommodate the variability of waste feedstocks and to understand the effects of feedstock characteristics on the final nanofiber properties. Here, lignocellulosic nanofibers with unique properties are produced from various waste biomass based on a simple and low-cost process using mild operating conditions. The process robustness is demonstrated by diversifying the feedstock, ranging from food crop waste (corn stover) to invasive grass species (reed canary grass) and industrial lignocellulosic residues (industrial hemp). This comprehensive study provides a thorough examination of the influence of the feedstocks’ physico-chemical characteristics on the conversion treatment, including process yield, degree of delignification, effectiveness of nanofibrillation, fiber morphology, surface charge, and density. Results show that nanofibers have been successfully produced from all feedstocks, with minor to no adjustments to process conditions. This work provides a framework for future studies to engineer nanocellulose with specific properties by taking advantage of biomass feedstocks’ intrinsic characteristics to enable versatile applications.

## 1. Introduction

Nanocellulose products are primarily produced from bleached wood pulp, a high-purity, expensive feedstock comprising virtually pure cellulose [1]. Current prices of nanocellulose products made of bleached pulp can reach up to US$1000/kg depending on the product characteristics [2,3]. Previous studies have shown that pulp feedstock represents one of the major operating costs of nanocellulose production processes. Hence, using lower-cost feedstock alternatives can significantly improve the economic feasibility of nanocellulose manufacturing [4,5] and make this nanomaterial more economically viable for large-volume applications such as bioplastics. In addition to high cost, nanocellulose produced from bleached pulp presents limited properties since it consists of a single biopolymer, cellulose. This linear, high molecular weight homopolymer mainly offers strength, crystallinity, rigidity, and hydrophilicity to the nanofiber product. If fibrils with other properties are desired, such as increased anionic surface charge or improved hydrophobicity, additional surface modification reactions must be carried out during the preparation or post-production of nanocellulose to change the nanofibers’ chemistry for specific applications [6].

There is increasing interest in using low-purity biomass waste feedstocks such as agricultural residues and forest residues, which contain other components besides cellulose (e.g., hemicellulose, lignin, pectin, ash, and/or extractives), to produce nanofibers due to their lower cost compared to bleached pulp [1,7]. Using waste feedstocks combined with mild processing conditions generates a unique type of nanocellulose product called lignocellulosic nanofibers, commonly containing the biopolymers cellulose, hemicellulose, and/or lignin [8]. To date, several methods to prepare lignocellulosic nanofibers have been reported in the literature, encompassing a variety of feedstocks and processing techniques. To list a few, common feedstocks used in the preparation of lignocellulosic nanofibers include unbleached pulp, thermomechanical pulp, wheat straw, sugarcane bagasse, among other agricultural residues; and the conversion process techniques may vary from purely mechanical (e.g., refining, homogenization, microfluidization, ultrasonication) to chemical treatments (pulping and bleaching reactions, acid hydrolysis, enzymatic hydrolysis, TEMPO oxidation, ionic liquids) and their combinations [7,9]. There is a preference for greener, milder, and less energy-intensive methods for reduced costs and environmental impacts [9], and the choice of feedstock and processing conditions may also dictate the type and amount of each biopolymer (cellulose, hemicellulose, and lignin) present in the final lignocellulosic nanofibers.

The intrinsic characteristics of each of the three biopolymers will influence the properties of the final product without requiring additional, and often expensive, post-processing modification reactions. Hemicelluloses are branched, low molecular weight heteropolysaccharides whose specific chemical composition varies with different plant species. Hemicelluloses commonly confer colloidal stability, easier fibrillation, and negative charge to nanofibers [8,10,11]. Lignin is an amorphous polymer that can present different structures depending on the feedstock and process used. It has been reported that lignin provides hydrophobicity, improved barrier properties, antimicrobial activity, and more to the nanofibers [8,12,13]. The use of waste feedstocks that are chemically heterogeneous offers considerable economic and sustainability benefits to nanocellulose production because they are much cheaper than conventional pulp [1,14], they do not require purpose-grown feedstock, and they can produce unique biomaterials with intrinsic properties inherited from the original biomass feedstock that can be modified for a targeted end use.

To use biomass waste feedstocks for nanocellulose production, robust conversion process technologies must be developed that can accommodate the impurities that come with such feedstocks. In addition, nanocellulose manufacturing processes should be feedstock-flexible, cost-effective, scalable, environmentally friendly, and able to operate standalone (i.e., not depending on integration with existing pulp and paper mills) to achieve commercialization [15]. In a previous study, our research group established a conversion process to produce lignocellulosic nanofibers from wheat straw biomass, a highly available food crop residue in the United States. The conversion process consists of mild alkaline peroxide pulping (for delignification) and peracetic acid pretreatment (for carbohydrate oxidation) followed by mechanical treatments (for fibrillation), generating unique lignocellulosic nanofibers with excellent bioplastic reinforcement properties [16]. The previous research demonstrated a promising route to producing inexpensive lignocellulosic nanofibers directly from waste feedstocks, motivating a further techno-economic analysis and life cycle assessment of the lignocellulosic nanofiber product. The economic and environmental assessment performed by our research group showed that the lignocellulosic nanofibers produced from wheat straw have a selling price lower than US$5/kg (dry basis) and have much lower life cycle CO_2_ emissions than nanofibers produced using state-of-the-art methods such as TEMPO oxidation [15]. Further investigation is now necessary to test the process’s feedstock flexibility using diverse waste feedstocks from plant species available in various locations. The present work will evaluate the robustness of our previously established process by using different types of feedstocks to produce lignocellulosic nanofibers. In this study, we selected three types of biomass waste feedstocks available in various regions across the United States, including another food crop residue (corn stover), an invasive grass species (reed canary grass), and an industrial lignocellulosic residue (industrial hemp).

Corn stover (CS) is an agricultural crop residue comprising the leftover stalks and leaves from corn production. CS is the largest source of agricultural residue in the United States, representing about 70% of the country’s total annual crop residue production, with availability estimated to reach up to 264 million dry tons by 2030 [17]. Even though the majority of CS residue is currently left on the fields, with some used in low-value applications such as animal bedding and cattle feed production [18], CS has also been the focus of several studies for biofuels and biochemicals production during the past years, including many investigations from the National Renewable Energy Laboratory (NREL) [19,20,21], due to its high availability and low price.

Reed canary grass (*Phalaris arundinacea* L.—RCG) is a lignocellulosic perennial crop that can grow on marginal lands unsuitable for food crops [22]. RCG is considered an invasive species in United States wetlands, causing a reduction in native plant diversity and requiring management strategies to control its spread [23,24]. RCG is primarily used in low-value agriculture applications such as hay production, straw or bedding for livestock, and soil conservation. Still, RCG has been studied for use in other applications, including the pulp and paper industry as a replacement for hardwood fibers and other energy conversion processes [22,25].

Industrial hemp (*Cannabis sativa* L.—IH) is a fast-growing, non-wood plant fiber crop with low water and nutrient requirements that grows in various environmental conditions [26,27]. While IH seeds are currently used for oil extraction, the remaining stalks (which represent about 70% of the plant’s dry weight) have been either used in low-value applications or have just been sent to waste [26]. Morphologically, IH stalks contain two types of fibers: bast (very long, about 10 to 20 times longer than fibers from hardwoods, softwoods, and agricultural residues) and core fibers (shorter fibers with similar physical characteristics to hardwood fibers) [25,28]. Bast fibers are commonly used in ropes, paper, textiles, and composites, while core fibers are used in paper, construction materials, biofuels, and others [27].

The three biomass feedstocks selected in this study (CS, RCG, and IH) have great potential for high-value biomaterial applications, either due to their high availability, invasive nature, low-value market, or low water and nutrient requirements. In this work, they are used to produce high-value lignocellulosic nanofibers via the conversion process previously described by our research group [16]. The present work aims to (1) assess the robustness of the conversion process by using agricultural waste feedstocks from various plant species and (2) elucidate how the specific chemical and physical characteristics of the biomass feedstocks affect the properties of the final products. It should be noted that our goal is not to determine which feedstock is best but rather to demonstrate that the conversion process employed is robust and can produce lignocellulosic nanofibers using various feedstocks. The investigation starts with assessing the complete chemical composition (including carbohydrates, lignin, extractives, and ash) of all biomass feedstocks before and after each chemical treatment. The recoveries of specific components (carbohydrates and lignin) at different process stages are calculated and compared. Then, lignocellulosic nanofibers produced from all feedstocks are characterized (optical transmittance of suspensions, surface chemistry, charge density, crystallinity, and morphology) and compared. Ultimately, the present study applies a comprehensive approach to understanding how we can engineer the properties of the final nanocellulose products for specific applications by taking advantage of the feedstock’s inherent characteristics and modifying specific process conditions.

## 2. Materials and Methods

### 2.1. Materials

Three biomass feedstocks were used in this study. Corn stover (CS) chopped to 6 mm particle size was sourced from Forest Concepts in Auburn, WA. Reed canary grass (RCG) bales were sourced from farms in Lewis County, WA, and cut into half-inch pieces using a hand pruner. Industrial hemp (IH) stalks were sourced from the Squaxin Island tribe, WA, and chopped to 2 mm particle size. All biomass feedstocks were air-dried and stored in plastic buckets until use.

The following chemicals were used for the reaction steps: 32% (*w*/*w*) peracetic acid (Sigma Aldrich, Saint Louis, MO, USA), 50% (*w*/*w*) sodium hydroxide (VWR Chemicals BDH), 50% (*w*/*w*) hydrogen peroxide (Cascade Columbia Distribution), diethylenetriamine pentaacetic acid (DTPA) 98+% (Acros Organics, Fisher Scientific, Hampton, NH, USA).

### 2.2. Alkaline Peroxide Pulping

The three biomass feedstocks underwent alkaline peroxide pulping following the same procedure previously reported by our group for the conversion of wheat straw [16]. After pulping, the samples were vacuum filtered, and the pulps were extensively washed with deionized (DI) water. Finally, the washed pulps were refined using a laboratory PFI mill for 30,000 revolutions, resulting in refined pulps.

### 2.3. Peracetic Acid (PAA) Pretreatment

The refined pulps were submitted to peracetic acid (PAA) pretreatment based on the procedure reported by [16] with some modifications. First, the refined pulps were mixed with PAA solution (2 wt.%) at pH 4.8 in a plastic bottle to achieve 5% pulp consistency, and the reaction was carried out at 85 °C for 45 min in a water bath. Due to IH’s different physico-chemical characteristics and delignification behavior compared to the other feedstocks (as seen in the results and discussion section), an additional PAA pretreatment condition was carried out for the IH sample with 4 wt.% PAA solution and 1% pulp consistency (resulting in a PAA charge approximately 10 times higher than the original reaction condition), producing an additional sample named IH 10×. All samples were vacuum filtered, and the PAA-treated pulps were thoroughly washed with 0.01 M NaOH followed by DI water.

### 2.4. Lignocellulosic Nanofibers Production

The different PAA-treated pulps were fibrillated under the same conditions using a blender (30 min) at 0.4 wt.% consistency, followed by homogenization with a horn ultrasonicator operated at 100% amplitude and 0.1 wt.% consistency for 4 min. The samples were then centrifuged (4500 rpm, 15 min) to separate two product fractions: supernatant consisting of lignocellulosic nanofibrils (LCNF) and precipitate consisting of lignocellulosic microfibrils (LCMF). In this work, the term lignocellulosic nanofibers will be used to refer to both LCNF and LCMF fractions in a general sense. The LCNF suspensions were concentrated by vacuum-rotary drum evaporation at 90 °C. All samples were stored in glass bottles at room temperature until use.

### 2.5. Characterization Techniques

#### 2.5.1. Chemical Composition

The chemical composition of untreated biomass feedstocks, alkaline peroxide pulps, and PAA-treated pulps was assessed. The carbohydrates, acidic/uronic acids, and lignin content (including both acid-soluble and acid-insoluble lignin) were quantified according to methods previously described by our research group [29,30,31,32]. Ash content was measured gravimetrically [33], and total extractives content was determined by water and ethanol Soxhlet extraction with a 12 h reflux time [34].

#### 2.5.2. Mass Yield and Component Recovery after Pulping and Pretreatment

The total mass yield after pulping and PAA pretreatment was determined gravimetrically by comparing the oven-dry (OD) mass of pulp obtained after each process with that of the untreated biomass, as seen in Equation (1).

Mass yield (%) = (Pulp mass after each reaction (g))/(Mass of untreated biomass (g)) × 100(1)

The recoveries of holocellulose (i.e., cellulose and hemicellulose) and lignin components after pulping and PAA pretreatment were calculated by correlating the mass yield and chemical composition at each stage to that of the untreated biomass, as seen in Equation (2).

Component recovery (%) = (Amount of component in pulp (%) × Mass yield (%))/(Amount of component in untreated biomass (%))(2)

#### 2.5.3. Product Separation Yield

The product separation yield was determined by centrifugation. Two fractions of products were obtained after centrifugation: supernatant LCNF and precipitate LCMF. The product separation yields, expressed as percentages, were calculated by the OD weight ratio of each product fraction to the pre-centrifugation suspension, as shown in Equation (3).

Separation yield (%) = (post-centrifugation LCNF or LCMF dry mass (g))/(pre-centrifugation LCNF + LCMF dry mass (g)) × 100(3)

#### 2.5.4. Lignocellulosic Nanofibers Characterization

Optical transmittance of aqueous lignocellulosic nanofibers suspensions at 0.2 wt.% concentration was conducted using UV/VIS/NIR Spectrophotometer in the visible region (from 400 to 800 nm) at a scan resolution of 1 nm. DI water was used as a blank. The optical light transmittance was evaluated using the percent transmittance at 660 nm [35].

The surface charge density of different lignocellulosic nanofibers was determined by conductometric titration based on the method described by Besbes et al. [36] with minor modifications. Briefly, 0.5 mL of 0.1 M HCl was added to 50 mL of 0.1 wt.% sample suspension and mixed for 10 min to protonate the carboxyl groups. Then, a titration was performed with 0.02 M NaOH at 100 µL increments, and the conductivity values were measured using a conductivity meter. The charge density (µmol COOH/g) was determined according to Equation (4), where V1 is the volume of NaOH required to neutralize the excess HCl, and the difference between V2 and V1 is the volume of NaOH used to neutralize the carboxylic acids.

Charge density = ((V2 − V1) × [NaOH])/(sample oven dry weight)(4)

Crystallinity index of different lignocellulosic nanofibers was determined by X-ray diffraction (XRD) using a Bruker D8 Discover coupled with a Pilatus 100K large-area 2D detector and a Cu Kα radiation generated at 50 kV and 1 mA. Diffractograms of neat films were taken over a 2ϴ angular range of 10–50° with 0.02° steps. The crystallinity index (CI) was calculated based on the Segal method, as shown in Equation (5):

CI (%) = (It − Ia)/It(5)

where It is the intensity of the crystalline peak (2 0 0) at 2ϴ = 22.7° and Ia is the intensity of the amorphous peak (1 1 0) at 2ϴ = 18° [37].

Specific functional groups within the different materials were characterized by Fourier-transform infrared spectroscopy (FTIR). Infrared spectra were analyzed using FT-IR Prestige-21 spectrometer (Shimadzu) coupled with a DLATGS detector attached to MIRacle ATR. The spectra were collected at ambient conditions in [550–4000] cm^−1^ range with a resolution of 4 cm^−1^ and from an accumulation of 40 scans. The spectra obtained were normalized by dividing all absorbance values by the largest absorbance value (i.e., based on the highest cellulose peak centered around 1026–1028 cm^−1^).

Nanofiber morphology was examined by scanning electron microscopy (SEM) and atomic force microscopy (AFM) techniques. AFM images of LCNFs were collected using a Bruker ICON AFM in contact mode and a scan rate of 1 Hz. LCNF suspensions were diluted to 0.001 wt.% with DI water and bath sonicated for 5 min to promote dispersion. An amount of 100 µL of LCNF was drop-casted onto a freshly cleaved mica disc previously coated with 50 µL of L-lysine, rinsed with DI water, and air-dried. The LCNF width and length were computed from at least 20 measurements of individual fibrils. SEM images of LCMFs were collected using an Apreo S (Thermofisher Scientific) coupled with a standard ETD in-chamber SE detector and a T2 in-column SE detector. LCMF suspensions were diluted to 0.01 wt.% with DI water and bath sonicated for 3 min; then, 10 µL of sample was drop-casted onto a clean SiO2 wafer. Before imaging, the samples were sputter-coated with a 4 nm thick platinum layer using a Leica EM ACE600 coater. Operating conditions were at high vacuum with an acceleration voltage of 2 kV and beam current of 6.3 pA. The LCMF width was computed from at least 20 measurements of individual fibrils. Unfortunately, it was not possible to measure LCMF fiber length due to the entanglement of fibers in the SEM imaging method.

## 3. Results and Discussion

**Figure 1:**
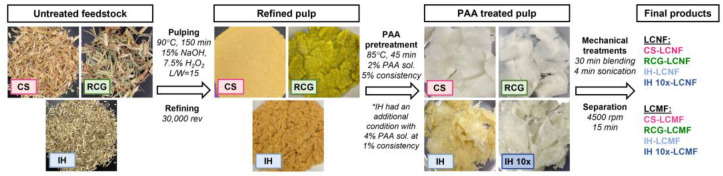
Summary of process steps to produce lignocellulosic nanofibers (LCNF and LCMF) from different biomass feedstocks. The same colors used in this figure to represent the different samples were used throughout the entire work. Particularly for IH biomass, an additional PAA pretreatment was performed at higher PAA loading, producing IH 10× sample.

Figure 1 summarizes the process steps to produce lignocellulosic nanofibers from CS, RCG, and IH. First, each biomass feedstock underwent alkaline peroxide pulping and refining, producing refined pulps. Then, the pulps underwent PAA pretreatment, generating PAA-treated pulps. In the specific case of IH, an additional PAA condition was carried out containing 10 times higher PAA charge during the reaction, producing IH 10× treated pulp. Finally, all PAA-treated pulps underwent mechanical fibrillation, homogenization, and separation, each generating two product fractions: lignocellulosic nanofibrils (LCNF) and microfibrils (LCMF). The chemical composition of each untreated biomass and their respective pulps was assessed after each reaction step, and the resulting lignocellulosic nanofibers (both LCNFs and LCMFs) were characterized (optical transmittance, surface chemistry, crystallinity, and morphology) and compared.

### 3.1. Effect of Chemical Treatments on Mass Yield and Chemical Composition

**Figure 2:**
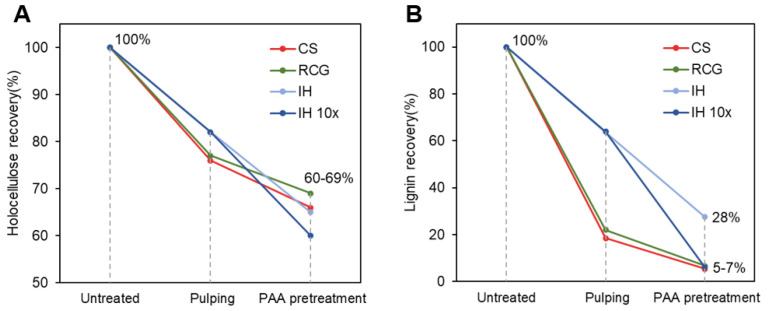
Recovery percentages of (**A**) holocellulose and (**B**) lignin components after pulping and PAA pretreatment related to untreated biomass. The complete recovery results are shown in Supporting Information, Table S2.

The effects of pulping and PAA pretreatment reactions on the main chemical components of the different biomass feedstock are represented in Figure 2. Figure 2A displays the recovery percentages of holocellulose (corresponding to both cellulose and hemicellulose fractions), and Figure 2B shows the recovery percentages of the lignin component. The total mass yield and complete chemical composition of each material are shown in Supporting Information, Table S1, and the complete holocellulose and lignin recoveries results are included in Supporting Information, Table S2. The goal of mild alkaline peroxide pulping was to partially remove lignin, enabling fibrillation while keeping most holocellulose intact for higher yields. Accordingly, Figure 2A shows that more than 76% of holocellulose was preserved in all feedstocks after pulping, demonstrating a low carbohydrate loss during the reaction. RCG had the lowest total mass yield (49%) after pulping compared to the other feedstocks due to its high extractives and ash content (Supporting Information, Table S1), which were removed during processing. The RCG mass yield could be improved further by using only the stem portion of the feedstock since the leaves contain the most ash and extractives [25].

Extensive delignification during pulping was observed for both CS and RCG, with similar delignification percentages ranging from 78–81% (i.e., 19–22% lignin recovery), while IH had the lowest delignification of only 36% (i.e., 64% recovery) (Figure 2B). The low delignification observed for IH is in accordance with a previous study by Wawro et al., where the authors reported little effect on the lignin content of IH fibers after mild NaOH treatment (90 °C for 5 h) [38]. The discrepancy in the extent of delignification of IH compared to the other feedstocks can be explained by its distinctive physical characteristics. IH stalks have a similar physical structure as woody biomass, and alkaline pulping treatments performed on IH biomass are typically carried out at much higher temperatures and pressure (typically 120–180 °C) [28,39], similar to the conditions used in hardwood pulping. The mild pulping condition employed in the present study (i.e., 90 °C, atmospheric pressure) only solubilized the highly reactive lignin in IH, such as phenolic α-O-4 linkages that are easier to break during pulping at lower temperatures [40]. CS and RCG, being less recalcitrant, showed a more extensive delignification under the mild pulping conditions. These outcomes are in accordance with previous studies from the literature, where Joachimiak et al. compared the pulping yields and delignification degree of a hardwood biomass (Birch sawdust) with that of a grass (Miscanthus stems) [41]. The authors found that, under the same pulping conditions, the grass displayed much higher and faster delignification than the hardwood, which was attributed to chemical and morphological differences between the two feedstocks.

After PAA pretreatment, all feedstocks displayed a trend of further delignification accompanied by minor carbohydrate losses, comparable to previous studies employing PAA treatments on different feedstocks [42,43,44]. These results demonstrate the high selectivity of PAA toward lignin and the process’s capacity to preserve high amounts of holocellulose (>60%) in the fibers. Generally, acid treatments cause extensive solubilization of the hemicellulose fraction from the fibers [45], lowering yields. In contrast, the mild PAA pretreatment used in this work successfully preserved hemicellulose in the final fibers, as seen by the presence of arabinan, galactan, xylan, and mannan in their composition (Supporting Information, Table S3). The recovery of hemicellulose in the final fibers is advantageous because it increases the total mass yields and provides unique properties to the final product, as discussed below.

Compared to the untreated biomass, both CS and RCG had lower lignin recoveries (5–7%), while IH still retained 28% of the original lignin in its composition (Figure 2B). These results reiterate that IH lignin is more difficult to remove than that of CS and RCG under the same mild reaction conditions, even in the presence of oxidizing chemicals such as PAA. Due to its higher residual lignin content, IH PAA-treated pulp presented a yellow-toned color, as shown in Figure 1. By increasing the amount of chemicals during PAA pretreatment of IH by 10×, a more substantial delignification was achieved (with up to 94% lignin removal), reaching similar recoveries to those obtained for CS and RCG under milder PAA dosages (Figure 2B). This improvement in delignification at higher PAA charges is consistent with previous reports in the literature, where increased PAA concentration during a bleaching reaction at pH 5 improved the whiteness index (indicating a lower lignin content) of IH pulp [46]. Interestingly, the tenfold increase in PAA chemical load mainly affected the lignin content (as seen by a 22% difference in the lignin recovery of IH 10× compared to IH), while only minor variations were observed in holocellulose (<5% difference). This outcome shows that the lignin component is more sensitive to changes in PAA load than the carbohydrates [47], which may again be attributed to high PAA selectivity toward lignin.

### 3.2. Nanofibers Characterization

#### 3.2.1. Suspension Optical Transmittance

**Figure 3:**
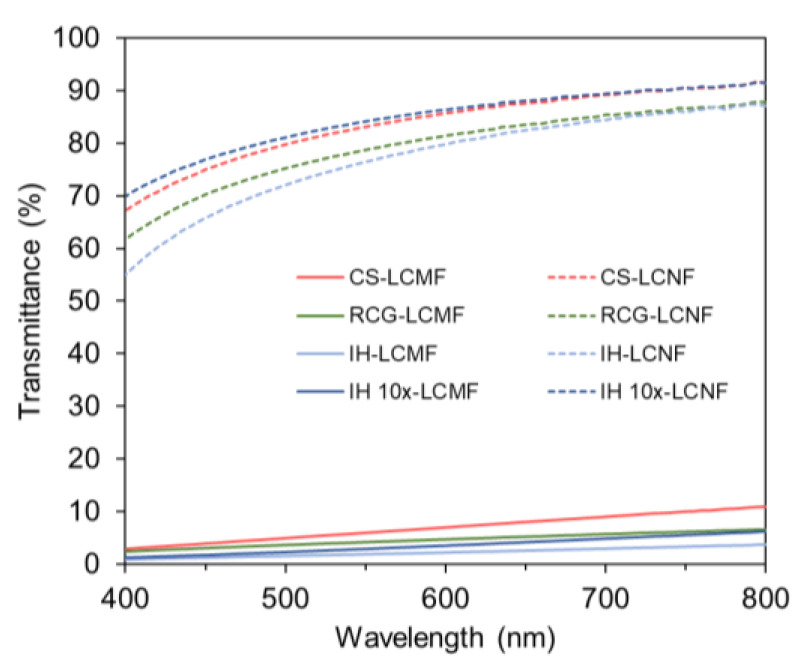
Optical transmittance spectra of different lignocellulosic nanofibers suspensions.

Figure 3 shows the complete light transmittance spectra of LCMF and LCNF suspensions obtained after the mechanical fibrillation and homogenization of PAA-treated pulps. Photographs of the different suspensions and their percent light transmittance values at 660 nm are included in Supporting Information, Figure S1. The mild process utilized in this study successfully produced gel-like suspensions of LCNF and LCMF from all feedstocks tested. As seen in Figure 3, all LCNF fractions exhibited high light transmittance values (83–88% at 660 nm), demonstrating the presence of very tiny nanofibrils, while all LCMF fractions showed a milky-white appearance and low transmittance values (3–8%) due to higher light scattering by the presence of larger fibrils [48,49]. In addition, all LCNFs and LCMFs demonstrated good colloidal stability regardless of the feedstock type. This is likely associated with the presence of hemicellulose heteropolysaccharides, as indicated by the arabinan, galactan, xylan, and mannan analyses in Supporting Information, Table S3. Hemicellulose is known to promote colloidal stability through steric hindrance and Coulombic repulsion [8]. IH LCNF and IH LCMF presented lower transmittance values across the entire spectra (Figure 3) as a result of their higher residual lignin content (12%) compared to the other samples (2–4%) (Supporting Information, Table S1). Lignin is well known to have relatively strong light absorption [49].

#### 3.2.2. Surface Chemistry

**Figure 4:**
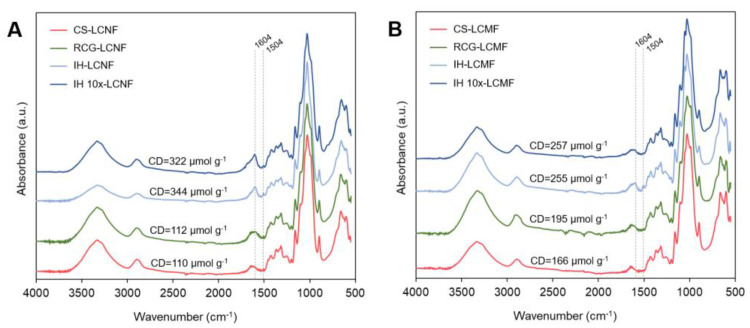
FTIR spectra showing specific chemical bonds of (**A**) LCNFs and (**B**) LCMFs of different biomass feedstocks. Charge density (CD) values obtained via conductometric titration are also shown (the titration curves are shown in Supporting Information, Figure S2).

Figure 4 shows FTIR spectra with specific bonds of LCNF (Figure 4A) and LCMF (Figure 4B) fractions obtained from each feedstock, along with the charge density (CD) values obtained by conductometric titration. It can be seen that both LCNFs produced from IH presented higher CD values (322–344 µmol g^−1^) than those from CS and RCG (110–112 µmol g^−1^) (Figure 4A). A similar trend was observed for LCMFs produced from IH, but to a lower extent (Figure 4B). The higher CD values for IH products were further verified by the presence of a more prominent peak at 1604 cm^−1^ in FTIR spectra of both IH-LCNF and IH 10×-LCNF compared to that of CS and RCG. The band at 1604 cm^−1^ has been previously associated with carboxyl groups present in hemicellulose [50,51] that promote a negative surface charge to the fibers. Furthermore, the peak at 1504 cm^−1^ corresponding to C=C stretching vibration of lignin aromatic rings [52] was more prominent in the IH-LCNF and IH-LCMF samples, which agrees well with the higher residual lignin content obtained for this sample.

When compared to nanofibers produced via harsher reactions such as TEMPO-mediated oxidation (usually around 1000 µmol g^−1^), the CD values reported in this work for CS, RCG, and IH (110–344 µmol g^−1^) are relatively lower due to the milder oxidation reaction that occurs during PAA pretreatment, as previously reported by our group [16]. Studies reported that CD largely improves nanofibrillation by promoting Coulombic repulsion forces between the fibers, hence the high efficacy of TEMPO oxidation in producing very small nanofibers [53]. Interestingly, despite the low CD values obtained in this work, LCNFs of comparable morphology to those obtained via harsher TEMPO oxidation were obtained (see Section 3.2.3 below) by means of milder and greener treatments. This outcome can be attributed to the high hemicellulose preservation achieved after both pulping and PAA pretreatment reactions, as previously discussed, and this biopolymer’s unique steric hindrance capabilities [8,10].

**Figure 5:**
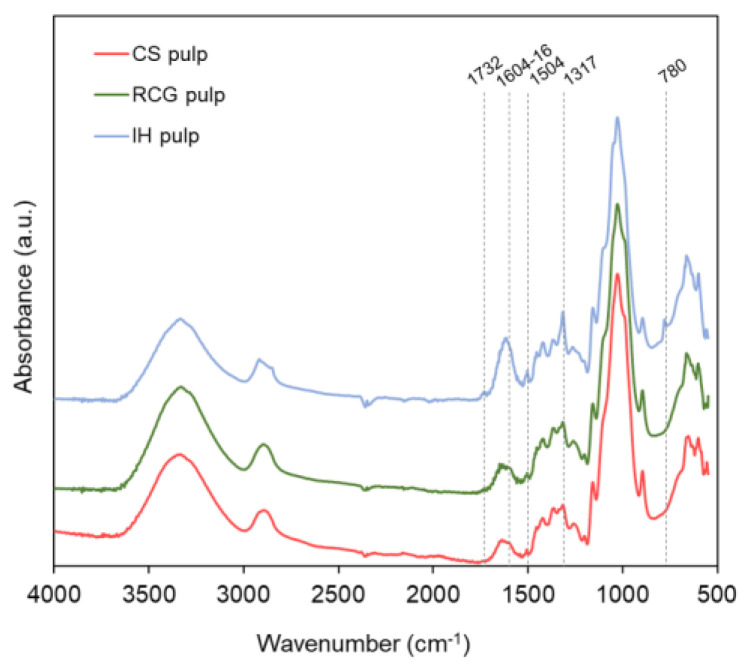
FTIR spectra showing specific chemical bonds of pulps of different biomass feedstocks.

To further elucidate the origin of the higher CD of both products made from IH biomass, FTIR spectra of the pulps after alkaline peroxide pulping (i.e., before PAA pretreatment) were also collected (Figure 5). This additional data will determine whether the carboxyl groups in IH products mainly originated during the oxidation reaction of PAA pretreatment or came from the original biomass. Interestingly, IH pulp again showed a much more prominent peak at 1604–16 cm^−1^ (which overlaps with water around 1630 cm^−1^) associated with the carboxyl groups of glucuronic acid in hemicellulose [50,51]. In addition, the peaks at 780 cm^−1^ and around 1732 cm^−1^ appeared exclusively in IH pulp, representing molecules that are inherent to this specific type of biomass. The band at 780 cm^−1^ has been previously assigned to the carboxyl groups of hemicellulose [54], while that around 1732 cm^−1^ has been attributed to C=O stretching vibration of either acetyl groups present in xyloglucan (a specific type of hemicellulose present in IH biomass) [51,55,56] or carboxylic ester groups in pectin [57,58].

The above results show that IH’s higher carboxyl content, compared to CS and RCG, is mainly associated with hemicellulose acid groups and the presence of pectins. The published literature observed that IH biomass has a high galacturonic acid content attributed to the presence of pectin molecules such as rhamnogalacturonan-I [59,60]. Correspondingly, the untreated IH biomass used in this study showed higher acetyl/uronic acids content (5.5%) compared to the other untreated feedstocks (3.1–3.8%) (Supporting Information, Table S1), confirming the presence of pectin substances in IH. Therefore, the higher CD obtained for LCNF and LCMF from IH is attributed mainly to IH biomass’s inherent hemicellulose and pectin compounds, with some posterior intensification during the oxidation reactions from PAA pretreatment. 

Finally, the FTIR peak at 1317 cm^−1^ has been assigned to C-O stretching of C5 substituted aromatic rings, such as syringyl and condensed guaiacyl units of lignin [50,51]. Although this peak is present for all three specimens, it is more prominent in IH-derived materials as a result of IH pulp’s higher lignin content (19%) compared to CS and RCG pulps (6 and 10%, respectively) (Supporting Information, Table S1).

#### 3.2.3. Fiber Structure and Morphology

Table 1 summarizes the separation yields of lignocellulosic nanofibers (LCNFs and LCMFs) after centrifugation and their structural and morphological characteristics, as determined by XRD, AFM, and SEM (i.e., crystallinity index, fibril width and length, and fibril aspect ratio). As seen in Table 1, similar product yields and morphology were obtained despite widely different feedstocks. Equivalent amounts of LCNF and LCMF fractions were obtained from all feedstocks, with LCNF yields ranging from 25–34% (and corresponding LCMF yields of 66–75%), demonstrating the unique feedstock-flexibility trait of the process. Among the three biomass feedstocks tested, IH and IH 10× samples had the lowest LCNF yields (27 and 25%, respectively), with correspondingly the highest LCMF yields (73% and 75%), suggesting the lower effectiveness of the mechanical treatments on IH biomass. Interestingly, the lignin content seemed to not play a crucial role in the extent of nanofibrillation of IH, as both IH and IH 10× resulted in comparable LCNF/LCMF separation yields. Remarkably, varying lignin contents between 3–12% (Supporting Information, Table S1) did not affect the extent of the release of nanofibrils from IH in the present process.

The lower LCNF yields of samples prepared from IH compared to CS and RCG demonstrate a lower degree of nanofibrils released from the bigger fibrils in the original hierarchical structure of IH. This outcome results from the physical structure of IH biomass, which comprises bast and core fibers. Bast fibers are incredibly long, about 25 mm in length (as a comparison, softwood cells are about 3.5 mm in length), while core fibers have similar physical characteristics as hardwoods, being 0.8 mm in length [28]. The diverse fiber sizes present in untreated IH biomass possibly reduced the effectiveness of the mechanical treatment, resulting in incomplete fibrillation and lower LCNF yields. 

The crystallinity index (CI) of the different lignocellulosic nanofibers is also included in Table 1. Little difference was observed between the CI of LCNF and LCMF fractions produced from the various biomass feedstocks (from 64% to 75%).

**Figure 6:**
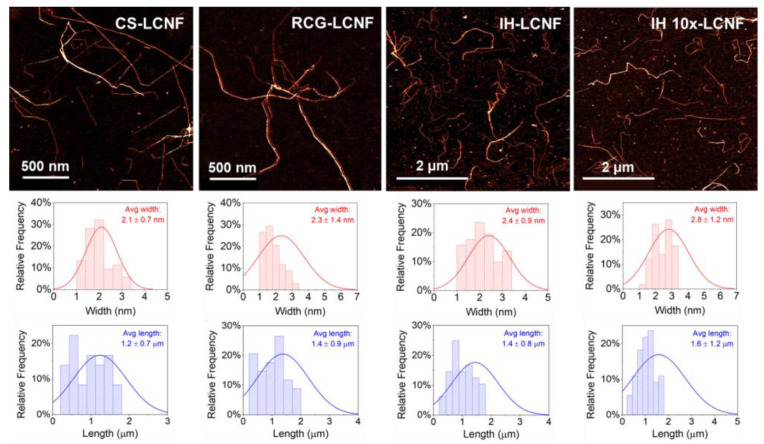
AFM images and size distribution curves of LCNF from different biomass feedstocks.

The morphology of the LCNF fractions generated from different biomass feedstocks was examined by AFM imaging (Figure 6), and the fibril dimensions are listed in Table 1. Representative AFM images revealed that all LCNFs had the morphology of elementary fibrils, with average widths ranging from 2.1 to 2.8 nm and lengths ranging from 1.2 to 1.6 μm. These similar morphological characteristics yielded LCNFs with comparable aspect ratios (i.e., 565–599) regardless of the nature of the feedstock (Table 1). In addition, all LCNFs obtained in this study presented sizes corresponding to nanofibrils prepared from high-purity hardwood pulp feedstock via harsher TEMPO oxidation pretreatment [53]. These results demonstrate the effectiveness of the present process in producing high quality LCNFs from a wide range of waste feedstocks and using milder reactions with minor to no process adjustments required. Surprisingly, despite their different lignin contents, both IH-LCNF and IH 10×-LCNF also had comparable LCNF morphologies (Figure 6), showing that lignin content did not have an effect on the morphology of the LCNF fraction obtained from IH biomass.

**Figure 7:**
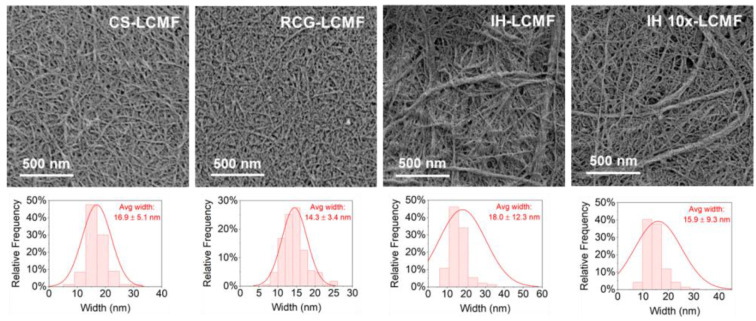
SEM images and size distribution curves of LCMF from different biomass feedstocks.

The morphology of the LCMF fractions was characterized by SEM imaging (Figure 7), and the fibril widths are included in Table 1. CS and RCG feedstocks produced more uniform LCMFs than IH (Figure 7). IH-LCMF and IH 10×-LCMF presented several fibril bundles, supporting the above interpretation of incomplete fibrillation during IH processing, and, therefore, resulted in the highest average widths and the broadest standard deviations (Table 1). LCMFs produced from IH also presented broader, right-skewed width distribution curves than those produced from CS and RCG (Figure 7). Interestingly, IH 10×-LCMF had a lower standard deviation than IH-LCMF, indicating that a lower lignin content reduced the occurrence of partially fibrillated fibrils in the LCMF fraction. Overall, the average width of the LCMFs produced in this study ranged between 14 and 18 nm independent of the type of feedstock used. The obtained fiber size is comparable to other lignocellulosic nanofibers prepared from various feedstocks and processes, where reported fiber widths varied from 6 nm up to around 100 nm, with most cases applying to the 10–30 nm range [9]. Particularly, the LCMFs produced from IH biomass exhibited a more heterogeneous size distribution comprising individual microfibrils and bundles.

## 4. Conclusions

The present study demonstrates that lignocellulosic nanofibers may be successfully produced from corn stover, reed canary grass, and industrial hemp via the same conversion process using mild conditions. The process was proven robust, generating products with similar morphologies despite widely different feedstocks and offering a practical pathway to manufacture lignocellulosic nanofibers from other agricultural waste biomass such as wheat straw, rice straw, rice husk, sugarcane bagasse, and switchgrass for example. This work also reported how the feedstocks’ physico-chemical characteristics influenced the final nanofibers’ properties. A feedstock with physical characteristics similar to woody materials (IH in this study) was more difficult to delignify under the mild reaction conditions, resulting in nanofibers with higher lignin recovery (28% recovery) compared to other feedstocks (5–7%); but increasing the chemical loading during PAA pretreatment resulted in higher delignification of IH (6% lignin recovery) with minor carbohydrate loss. IH’s unique physical structure (comprising bast and core fibers of vastly different sizes) also affected the efficacy of the mechanical treatment step, impacting the nanofibers’ separation yields and morphology. Finally, feedstocks with large amounts of glucuronic acids in hemicellulose and/or pectins produced nanofibers with greater anionic surface charge (up to three times higher charge density than from other feedstocks).

Ultimately, using waste biomass feedstocks instead of bleached pulp enables engineering the nanofiber properties due to the presence of cellulose, hemicellulose, and lignin, where each can provide distinctive properties to the final product. We use nature’s inherent characteristics to generate nanofibers with specific properties instead of expensive post-processing surface modification reactions. The present process also allows for customization of the nanofiber properties by tuning the reaction condition parameters. In the long run, using low-cost waste feedstocks available in different regions of the United States can provide substantial economic and sustainability benefits to nanocellulose production, presenting a significant stride toward large-scale production and commercialization for various applications.

## Figures and Tables

**Table 1:** Separation yields of LCNF and LCMF fractions after centrifugation and their structural and morphological characteristics (crystallinity index, fibril width and length, and fibril aspect ratio).

		Separation Yield (%)	CI (%)	Fibril Width (nm)	Fibril Length (μm)	Fibril Aspect Ratio
LCNF	CS	30	71	2.1 ± 0.7	1.2 ± 0.7	585
	RCG	34	66	2.3 ± 1.4	1.4 ± 0.9	591
	IH	27	64	2.4 ± 0.9	1.4 ± 0.8	599
	IH 10×	25	66	2.8 ± 1.2	1.6 ± 1.2	565
LCMF	CS	70	72	16.9 ± 5.1	-	-
	RCG	66	69	14.3 ± 3.4	-	-
	IH	73	72	18.0 ± 12.3	-	-
	IH 10×	75	75	15.9 ± 9.3	-	-

## Data Availability

Not applicable.

## Supplementary Materials

The following supporting information can be downloaded at: https://www.mdpi.com/article/10.3390/polym15040937/s1. Figure S1: Photograph of the suspensions and their respective percent transmittance at 660 nm; Figure S2: Conductometric titration curves of (A) LCMF and (B) LCNF from different feedstocks; Table S1: Total mass yield and chemical composition of untreated biomass feedstocks, alkaline peroxide pulps, and PAA-treated pulps of corn stover (CS), reed canary grass (RCG), and industrial hemp (IH). In the special case of IH, one additional PAA reaction condition was studied employing about 10 times higher PAA charge; Table S2: Holocellulose and lignin recovery after alkaline peroxide pulping and PAA treatment related to the untreated biomass; Table S3: Relative carbohydrate composition in holocellulose of untreated biomass, alkaline peroxide pulps, and PAA-treated pulps of the different biomass feedstocks.
