# Supplementary material for: A Robust Process to Produce Lignocellulosic Nanofibers from Corn Stover, Reed Canary Grass, and Industrial Hemp
Source: Polymers (Basel). 2023 Feb 14;15(4):937. doi: 10.3390/polym15040937 (PMC9967869; doi:10.3390/polym15040937)
Supplement: Supplementary file 1 [file polymers-15-00937-s001.zip › polymers-2162012-supplementary.pdf]

Supporting Information

A robust process to produce lignocellulosic nanofibers from corn stover, reed canary grass, and industrial hemp

Danielle Uchimura Pascoli [1,2,], Anthony Dichiara [1], Rick Gustafson [1] and Renata Bura [1,]*.*

*Corresponding authors

[1] School of Environmental and Forest Sciences, University of Washington, Seattle, WA 98195, USA

[2] VERDE Nanomaterials Inc. Davis, CA 95618, USA

*Correspondence: dpascoli@verdenano.com (D.U.P.); renatab@uw.edu (R.B.)

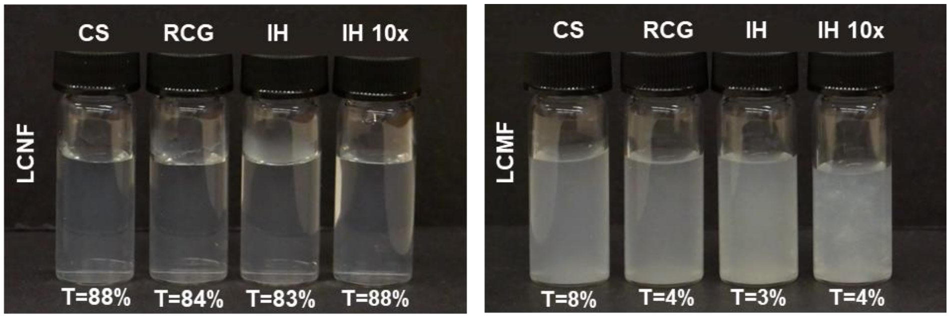

Figure S1. Photograph of the suspensions and their respective percent transmittance at 660 nm.

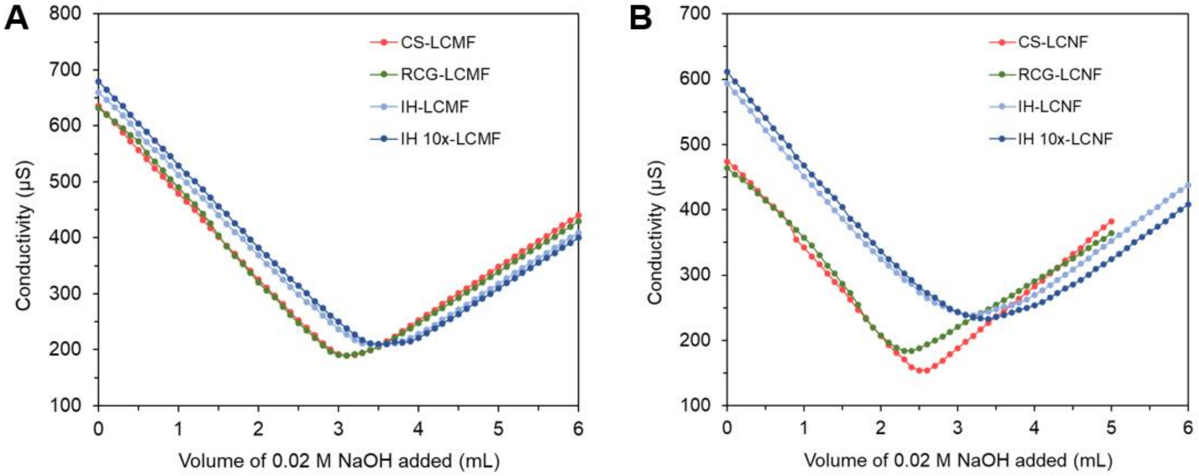

Figure S2. Conductometric titration curves of (A) LCMF and (B) LCNF from different feedstocks.

Table S1. Total mass yield and chemical composition of untreated biomass feedstocks, alkaline peroxide pulps, and PAA treated pulps of corn stover (CS), reed canary grass (RCG), and industrial hemp (IH). In the special case of IH, one additional PAA reaction condition was studied employing about 10 times higher PAA charge.

			Chemical composition (% of oven-dry weight)				
		Total mass yield (%)	Holocellulose [a] %	Uronic/acetic acids %	Lignin %	Ash %	Extractives %
Untreated	CS	-	71.9 ± 2.1	3.8 ± 0.2	21.3 ± 0.2	3.4 ± 0.1	7.8 ± 0.1
	RCG	-	50.4 ± 0.4	3.1 ± 0.1	21.5 ± 0.2	8.2 ± 0.1	29.6 ± 0.1
	IH	-	55.5 ± 0.2	5.5 ± 0.2	19.3 ± 0.2	5.2 ± 0.0	16.5 ± 0.3
Pulp	CS	60.6	90.3 ± 0.3	0.0 ± 0.0	6.5 ± 0.2	1.3 ± 0.2	2.1 ± 0.5
	RCG	49.0	79.5 ± 0.3	0.0 ± 0.0	9.7 ± 0.2	2.3 ± 0.1	4.2 ± 0.1
	IH	64.4	70.7 ± 3.4	0.0 ± 0.0	19.1 ±0.1	3.5 ± 0.2	3.0 ± 0.3
PAA treated pulp	CS	50.2	95.0 ± 0.3	0.0 ± 0.0	2.3 ± 0.3	< 0.1	NM
	RCG	40.0	86.7 ± 2.5	0.0 ± 0.0	3.6 ± 0.1	0.2 ± 0.0	NM
	IH	44.2	81.3 ± 0.5	0.0 ± 0.0	12.0 ± 0.2	2.2 ± 0.1	NM
	IH 10x	36.2	92.7 ± 1.0	0.0 ± 0.0	3.1 ± 0.1	1.5 ± 0.0	NM

NM = Not measured

[a] For detailed carbohydrate composition of holocellulose, see Supporting Information, Table S3

Table S2. Holocellulose and lignin recovery after alkaline peroxide pulping and PAA treatment related to the untreated biomass.

		Holocellulose recovery (%)	Lignin recovery (%)
Pulp	CS	76	19
	RCG	77	22
	IH	82	64
PAA treated pulp	CS	66	5
	RCG	69	7
	IH	65	28
	IH 10x	60	6

Table S3. Relative carbohydrate composition in holocellulose of untreated biomass, alkaline peroxide pulps, and PAA treated pulps of the different biomass feedstocks.

		Arabinan (%)	Galactan (%)	Glucan (%)	Xylan (%)	Mannan (%)
Untreated	CS	3.4	1.1	58.5	37.0	0.0
	RCG	5.7	2.5	60.7	31.0	0.0
	IH	2.3	3.5	70.8	21.2	2.2
Pulp	CS	2.3	0.4	64.7	32.6	0.0
	RCG	5.3	1.4	62.7	30.6	0.0
	IH	0.7	1.7	72.3	23.6	1.6
PAA treated	CS	2.3	0.3	66.1	31.4	0.0
	RCG	2.4	0.5	69.7	27.4	0.0
	IH	0.3	0.9	71.8	25.7	1.3
	IH 10x	0.3	0.8	72.1	24.6	2.2
